# Decreased Phenotypic Susceptibility to Etravirine in Patients with Predicted Genotypic Sensitivity

**DOI:** 10.1371/journal.pone.0101508

**Published:** 2014-07-07

**Authors:** Eva Agneskog, Piotr Nowak, Catharina Maijgren Steffensson, Maria Casadellà, Marc Noguera-Julian, Roger Paredes, Clas F. R. Källander, Anders Sönnerborg

**Affiliations:** 1 Division of Clinical Microbiology, Department of Laboratory Medicine, Karolinska Institutet, Stockholm, Sweden; 2 Unit of Infectious Diseases, Department of Medicine Huddinge, Karolinska Institutet, Stockholm, Sweden; 3 Cavidi AB, Uppsala, Sweden; 4 Institut de Recerca de la SIDA irsiCaixa, Hospital Universitari Germans Trias I Pujol, Universitat Autònoma de Barcelona, Catalonia, Spain; Burnet Institute, Australia

## Abstract

**Background:**

A sensitive, phenotypic reverse transcriptase (RT)-based drug susceptibility assay for the detection of etravirine (ETR) resistance in patient isolates was developed and compared with the results from direct sequencing and ultra-deep pyrosequencing (UDPS).

**Methods:**

Samples were obtained from 15 patients with antiretroviral therapy (ART) failure and from five non-nucleoside reverse transcriptase inhibitor (NNRTI)-naïve patients of whom four were infected by an NNRTI-resistant strain (transmitted drug resistance, TDR). In five patients, two consecutive samples (a and b) were taken for follow up of the virological response. HIV-1 RT was purified and drug susceptibility (IC_50_) to ETR was estimated. Direct sequencing was performed in all samples and UDPS in samples from nine patients.

**Results:**

Increased IC_50_ to ETR was found in samples from 13 patients where direct sequencing predicted resistance in only four. UDPS identified additional (N = 11) NNRTI resistance associated mutations (RAMs) in six of nine tested patients. During early failure, IC_50_ increases were observed in three of six patients without any ETR-RAMs detected by direct sequencing. In further two patients, who stopped NNRTI before sampling, increased IC_50_ values were found shortly after, despite absence of ETR-RAMs. In two patients who had stopped NNRTI for >1 year, a concordance between phenotype and genotypes was found. Two patients with TDR had increased IC_50_ despite no ETR-RAMs were detected by direct sequencing. UDPS revealed additional ETR-RAMs in four patients with a discrepancy between phenotype and direct sequencing.

**Conclusions:**

The RT-based phenotypic assay showed decreased ETR susceptibility in patients where direct sequencing predicted ETR-sensitive virus. This increased phenotypic sensitivity was to a large extent supported by UDPS and treatment history. Our method could be valuable for further studies on the phenotypic kinetics of NNRTI resistance. The clinical relevance remains to be studied in larger patient-populations.

## Introduction

The first generation non-nucleoside reverse transcriptase inhibitors (NNRTIs) nevirapine (NVP) or efavirenz (EFV) are frequently used in combination with two nucleoside reverse transcriptase inhibitors (NRTIs) as first-line antiretroviral therapy (ART) in both high- and low-middle income countries. The second generation NNRTI etravirine (ETR) is approved for treatment-experienced HIV-1 infected adults with resistance to NVP and/or EFV [1]–[3], although access to ETR in low-middle income countries (LMIC) is limited. ETR can be used in such patients due to its limited cross-resistance pattern relative to first generation of NNRTI. However, the pattern of resistance-associated mutations (RAMs) has to be considered and several constellations of mutations cause indeed cross-resistance [4], [5]. A large number of ETR RAMs have been revealed [6], including the major mutations L100I, K101E/P, E138A/G/K/Q, Y181C/I/V, Y188L, G190A/S/E, and M230L.

The genotypic resistance tests (GRT) used in clinical care detect RAMs that are present in >20% of the viral population [7]–[9]. More sensitive techniques, such as allele-specific PCR (ASPCR) [10]–[12] and ultra-deep sequencing [13], [14], are not yet used in the clinical routine. It has however been reported that minor resistant quasispecies may influence the outcome of ART [15]–[17]. One alternative to GRT is phenotypic tests which have several disadvantages in the clinical setting. However, new methods for reverse transcriptase (RT) extraction and sensitive RT assays have made analyses of drug susceptibility profiles of retroviral RTs recovered directly from plasma virions feasible [18]–[20]. Drug susceptibility testing on RT offers advantages compared to traditional phenotypic tests, such as short turnover time, lower cost, a need of only serology laboratory equipment, and they might therefore be useful in LMIC. This method has successfully been used to describe drug resistance to NVP [21] and also recently to ETR [22]. In contrast to genotypic assays, no complex interpretations are required.

The aim of the study was to thoroughly evaluate clinical samples in our newly adapted RT-based assay for assessment of resistance to ETR and to compare the obtained phenotype with the genotype obtained with standard direct sequencing. Also, we performed ultra-deep pyrosequencing (UDPS) to identify any minor sequence variants in the HIV-1 RT gene of cases where there was a discrepancy between the major genotype and the RT phenotype.

## Methods

### Patients

Altogether, 25 plasma EDTA samples of 20 HIV-1 infected patients were retrospectively included from the HIV cohort at Department of Infectious Diseases, Karolinska University Hospital, Stockholm, Sweden (Table 1). Of these, 15 treatment-experienced patients were randomly selected among subjects with ART failure. All of these patients had been treated or were treated with nevirapine or efavirenz. In patients 3, 5, 8, 9 and 11 (Table 1), who did not respond to treatment two consecutive samples (a and b) were drawn for follow up of the virological response. For eight samples (no 3b, 5a, 5b, 8a, 8b, 10, 11a, 18), the failing regimen contained an NNRTI. For 12 samples (no 1, 3a, 6, 7, 9a, 9b, 11b, 12, 14, 15, 17, 20), the NNRTI had been stopped earlier and the failing regimen contained now antiretroviral drugs from other categories (Table 1). Four treatment naïve patients (no 2, 13, 16, 19) were included since they had been infected with NNRTI-resistant strains. GRT had been performed within the clinical care by routine direct sequencing on samples from all of these individuals and one or more NNRTI mutations had been found in all of them, except in patient 7 (Table 2). One NNRTI-naïve patient (no 4), who had a K103R mutation, was chosen as a negative control since this mutation is reported not to affect ETR susceptibility. All the work presented in the manuscript was conducted in accordance with the Declaration of Helsinki. Ethical permission was obtained from the Regional Ethical Committee at Karolinska Institutet, Dnr. 2005/1167-31/3, Dnr. 2005/772-31/4 and written informed consent was obtained from each individual.

**Table 1:** Characteristics of 20 HIV-1 infected patients included in the study.

Pat.*	Sex*	Age*	Sub- type	Ongoing NNRTI treatment**	Earlier NNRTI treatment***	Other ongoing drugs****	HIV load (copies/ml)	CD4 count (cells/µl)
1	m	37	D	no	EFV (−134), NVP (−6)	no	17800	200
2	m	43	B	no	no	no	292000	100
3a	m	41	B	no	EFV (−472)	no	161000	10
3b				ETR (111)	EFV (−585)	DRV/r	117000	10
4	m	52	B	no	no	no	63000	373
5a	m	61	B	EFV (236)	-	LPV/r, T20	21800	300
5b				EFV (248)	-	LPV/r, T20	47000	335
6	f	48	C	no	EFV (−250), NVP (−60)	TDF, FTC, T20	6700	155
7	m	56	C	no	EFV (−286)	ATV,TDF,ABC,3TC	1500000	22
8a	m	46	B	NVP (337)	EFV (−418)	ABC, 3TC, TDF	13100	126
8b				NVP (339)	EFV (−420),	ABC, 3TC, TDF	3920	126
9a	m	61	B	no	EFV (−422), NVP (−192)	LPV/r, 3TC, RAL	8400	209
9b				no	EFV (−448), NVP (−218)	LPV/r, 3TC, RAL	20000	246
10	m	49	B	NVP (262)	EFV (−286)	ABC,3TC,TDF,T20	10400	662
11a	f	49	A	EFV (180)	-	ABC, 3TC	2600	260
11b				no	EFV (−308)	no	12200	219
12	m	42	C	no	NVP (−18)	ATV/r, ABC, 3TC	1100	380
13	f	40	C	no	no	no	151000	9
14	m	53	B	no	EFV(−172)	no	100001	420
15	m	51	B	no	EFV (−308)	DRV/r, TDF, FTC	910000	40
16	f	36	A	no	no	no	4420	303
17	f	42	D	no	NVP (−19)	ABC, 3TC, ZDV	1900	159
18	m	49	D	NVP (11)	EFV (−220)	ABC, 3TC, ZDV	12000	345
19	m	49	B	no	no	no	29000	234
20	f	49	B	no	EFV (−8)	no	4700	400

*Patients were selected due to a failing ART with the exception of patients no 2, 13, 16, 19 who were infected with an NNRTI-resistant strain and a treatment naïve patient no 4 who had the K103R mutation; a and b indicate a first and a second sample; m: male; f: female; age: years;

**Figure within brackets indicate the number of weeks from the start of the last ongoing NNRTI-treatment to the sampling date.

***Figure within brackets indicates the number of weeks from cessation of the prior NNRTI-containing treatment to the sampling date.

****ABC: abacavir; 3TC: lamivudine; FTC: emitricitabine; TDF: tenofovir; ZDV: zidovudine: LPV/r: lopinavir/ritonavir; ATV/r: atazanavir/ritonavir: DRV/r: darunavir/ritonavir; RAL: raltegravir; T20: enfuvirtid.

**Table 2:** Effects of etravirine on HIV-1 reverse transcriptase, recovered from plasma of HIV-1 infected patients.

			Mutations at amino acid											Antiviral score according to		
Patient/Sample	Fold difference in ETR IC_50_	90 V	98 A	100 L	101 K	103 K	106 V	108 V	138 E	179 V	181 Y	190 G	238 K	Stanford	Tibotec	Monogram
BH10*	1.0													NA	NA	NA
L100I*	12.0													15 (i)	2.5 (i)	4 (r)
1	11.8		S				A							0 (s)	0 (s)	2 (s)
2	>100					N			Q				T	10 (s)	1 (s)	2 (s)
3a	4.5		S							D				10 (s)	1 (s)	1 (s)
3b	>100	I	S							D	C			45 (i)	4.5 (r)	6 (r)
4	3.9					R								0 (s)	0 (s)	1 (s)
5a	45.8				I/R		G							0 (s)	0 (s)	0 (s)
5b	71.4				I/R		G							0 (s)	0 (s)	0 (s)
6	>100	I		I		N								20 (i)	3.5 (i)	5 (r)
7	0.7													0 (s)	0 (s)	0 (s)
8a	>100					N					C			30 (i)	2.5 (i)	4 (r)
8b	>100					N					C			30 (i)	2.5 (i)	4 (r)
9a	4.6		S			N		I					T	0 (s)	0 (s)	1 (s)
9b	9.2		S			N		I					T	0 (s)	0 (s)	1 (s)
10	13.6					N		I						0 (s)	0 (s)	0 (s)
11a	7.3					N				I				0 (s)	0 (s)	1 (s)
11b	4.1									I				0 (s)	0 (s)	1 (s)
12	2.0					N								0 (s)	0 (s)	0 (s)
13	68.8					N						A		10 (s)	1 (s)	1 (s)
14	29.1		S			N			A					5 (s)	1.5 (s)	3 (s)
15	2.9					N								0 (s)	0 (s)	0 (s)
16	1.7					N								0 (s)	0 (s)	0 (s)
17	2.9					N								0 (s)	0 (s)	0 (s)
18	4.1	I				N								0 (s)	1 (s)	1 (s)
19	6.4					N								0 (s)	0 (s)	0 (s)
20	7.2					N								0 (s)	0 (s)	0 (s)

*Two recombinant reverse transcriptase, (BH10)-wild-type (WT) and its mutant form L100I, were used as references. Based on the IC_50_ value of BH10-wild-type (1.0±0.18 µM), which was included in every run, the IC_50_ value and the fold-difference in susceptibility of each isolate were determined. A fold-difference of <5 was considered as sensitive, between 5–15 as decreased susceptibility, and above 15 as resistant.

Abbreviations: NA, not applicable; IC_50_, inhibitory effect; ETR etravirine.

**Genotype result was obtained six months earlier. The results of three genotypic scoring systems (Stanford, Monogram Weighted Score, Tibotec Weighted Genotype Score) predicting ETR susceptibility are presented. s: sensitive; i: intermediate; r: resistant. A Stanford score of 0-<15, ≥15-<60 and ≥60 are defined as susceptible, intermediate and resistant. Tibotec weighted genotypic score of 0–2, 2.5–3.5 and ≥4 are predictive of susceptible, intermediate and reduced response. Monogram defines a weighted score of 0–3 as susceptible and ≥4 as resistant.

### Chemicals

ETR (4-[[6-amino-5-bromo-2[(4-cyanophenyl)amino]-4-pyrimidinyl]oxy]-3.5-dimethylbenzonitrile) was a generous gift from Tibotec, Mechelen, Belgium. The drug was dissolved in dimethylsulfoxide (Sigma, St. Louis, MO) [22].

### Phenotypic RT-based analysis of resistance to ETR

Lysates containing intraviral RT were recovered from plasma using ExaVir Load, version 3 (Cavidi, Uppsala, Sweden) [18]. Briefly, virions from up to 1 ml plasma were bound to an ion exchange gel, washed, and the RT was extracted by lysing the purified virions. The RT was used to catalyse the incorporation of bromodeoxyuridine triphosphate (BrdUTP) into DNA via a poly-rA (ribodenylic acid template) plate. The amount of BrdU monophosphate (BrdUMP) incorporated into DNA was detected with an alkaline phosphatase (Ap) conjugated anti-BrdU monoclonal antibody [20]. The Ap substrate para-nitrophenyl-diphosphate was used for colorimetric detection. The susceptibility of the RT activity to ETR was measured using ExaVir Drug susceptibility assay, as previously described [22]. Briefly, this assay measures the ability of RT to catalyse the above reaction in the presence of a 2.5 fold step dilution of ETR. The RT activity at each drug concentration was recalculated into percentage of RT activity in the absence of drug. The IC_50_ values were calculated using the median effect equation [23]. The analysis was performed blindly in relation to the genotype results and to type of treatment. We have earlier described low IC_50_ values (mean ±SD: 2.5±1.0 µM) for RT activity in the presence of ETR in treatment-naïve patients and for reference recombinant RTs [22]. Also, the reproducibility analysis of the assay showed an inter-assay variation (CVs) of 9.4 and 11.1% and that the IC_50_ values for ETR (mean ± SD: 1.6±0.03 mM and 3.1±0.04) were not influenced by the RT amount within the 40–828 fg/ml RT range. In the present study, two reference recombinant RTs, BH10-wild-type and its mutant form L100I, were analysed together with patient samples in each assay. The results of the phenotypic tests were evaluated independently by two persons (E.A, C.FR.K).

### Scoring of ETR susceptibility

Three genotypic scoring systems were used to predict ETR susceptibility (Stanford University, Monogram Weighted Score, Tibotec Weighted Genotype Score). The Monogram and Tibotec systems use a score calculated from a list of mutations, each with their own weight factor [24]. The Stanford scores were obtained using http://hivdbstanford.edu/cgi-bin/Marvel.cgi. The correlation between Monogram/Tibotec/Stanford scorings and IC_50_ determined by our phenotypic RT assay was assessed by Spearman's rank test using software in GraphPad Prism (San Diego, California, USA).

### Direct HIV-1 sequencing

Sequencing of the HIV *pol* gene had earlier been performed within the clinical routine at the Department of Clinical Microbiology, Karolinska University Hospital, Stockholm. Samples were analysed using ViroSeq HIV-1 Genotyping System v2.0 (Abbott Diagnostics, Illinois, USA) according to manufacturer's protocol on an ABI PRISM 310 Genetic analyzer (Applied Biosystems).

### Ultra deep HIV-1 sequencing

HIV-1 RNA was extracted from 1 mL of plasma from nine samples (QIAamp Viral RNA Mini Kit, QIAGEN, Valencia, CA) after centrifugation at 9,000×*g* for 1 h. The 3′ end of the reverse transcriptase region was reverse transcribed and amplified using SuperScript III and Platinum Taq High Fidelity (Life Technologies, Paisley, UK). Primer used were Forward primer (HXB2: 2508–2528) 5′-GGA AGA AAT CTG TTG ACT CAG-3′ and Reverse primer (HXB2: 3441–3459) 5′- GAA GCA GAG CTA GAA CTG G-3′. Cycling conditions were: 30 min at 52°C for reverse transcription;2 min at 94°C for initial denaturation; 20 cycles of 2 min at 94°C, 30 sec at 50°C and 1 min 30 sec at 68°C; and a final polymerisation step of 5 min at 68°C. Amplicon libraries were generated from first-round PCR products. These amplicons incorporated adapters A and B, and also identifiers used in parallel sample sequencing needed for bidirectional 454 sequencing. Nested PCR conditions were a first denaturation step of 2 min at 94°C; 20 cycles of 2 min at 94°C, 30 sec at 50°C and 45 sec at 68°C; and a final polymerisation step of 3 min at 68°C using Platinum Taq High Fidelity (Life Technologies, Paisley, UK). A set of 5 overlapped amplicons were designed to cover the protein region previously amplified. Amplicon 1: Forward (2541–2566) 5′-TTA AAT TTT CCC ATT AGT CCT ATT GA-3′ and Reverse (2873–2890) 5′- ACT GGA TGT GGG TGA TGC-3′; Amplicon 2: Forward (2642–2667) 5′-AAA AGC ATT AGT AGA AAT TTG TAC AG-3′ and Reverse (2929-2951) 5′-ATA CTG CAT TTA CCA TAC CTA GT-3′; Amplicon 3: Forward (2780–2802) 5′-CAG AGA ACT TAA TAA GAG AAC TC-3′ and Reverse (3155–3174) 5′-AGA GGA ACT GAG ACA ACA TC-3′; Amplicon 4: Forward (2819–2839) 5′-TCA ATT AGG AAT ACC ACA TCC-3′, Reverse (3243–3265) 5′-TAT GAA CTC CAT CCT GAT AAA TG-3′; Amplicon 5: Forward (3018–3038) 5′- CCA GCA ATA TTC CAA AGT AGC-3′ and Reverse (3402–3422) 5′- GGA ACC AAA GCA CTA ACA GAA-3′.

Nested PCR products were purified using AMPure Magnetic Beads (Beckman Coulter, Inc, Brea, CA) to eliminate primer-dimers. Concentration and quality of purified PCR products was determined using fluorometry (PicoGreen, Life Technologies, Paisley, UK) and spectrophotometry (Lab on a Chip, Agilent Technologies, Foster City, CA). Equimolar amplicon pools were made to perform emPCR, adding a ratio of 1∶1 between molecules and 454-beads. Sequencing platform used was Genome System Junior (Life Sequencing/Roche). 454 analyses were performed with the 454/Roche proprietary AVA software v2.7 after checking for cross-sample and pNL4-3 contamination. The threshold level for detection of minority variants was set at 0.5%. Access to the viral sequences of the patients can be obtained after request to the author. The dataset have been deposited in the NIH/SRA repository with the accession number PRJNA246769.

## Results

### Phenotypic assay results in relation to mutational patterns

The two reference recombinant RTs; BH10-wild-type and its mutant form L100I, showed the expected IC_50_ values of 1.0±0.18 µM and 12.0±2.3 µM, respectively. RT was isolated from 20 plasma samples from 15 patients with treatment failure (Table 2). Based on the IC_50_ value of BH10-wild-type, which was included in every run, the IC_50_ value and the fold-change in susceptibility of each isolate were determined. A fold-change of <5 was considered as sensitive, between 5–15 as decreased susceptibility, and above 15 as resistant. In ten samples (3a, 4, 7, 9a, 11b, 12, 15, 16, 17, 18) with the lowest IC_50_ values and a fold change of <5, which were considered sensitive by our phenotypic assay, (mean ±SD: 3.1±1.3; range: 0.7–4.5 µM), there was a good concordance with the GRT. Thus, sequence analysis showed no mutations or non-ETR RAMs (3a: A98S + V179D; 4: K103R; 9a: A98S + K103N + V108I + K238T; 11b: V179I; 12, 15, 16 and 17: K103N; 18: V90I + K103N).

Six samples had IC_50_ values corresponding to a 5–15 fold decrease in susceptibility for ETR (range: 6.4–13.6 µM; 1: 11.8 µM, 9b: 9.2 µM, 10: 13.6 µM, 11a: 7.3 µM, 19: 6.4 µM, 20: 7.2 µM). In all of them (1: A98S + V106A; 9b: A98S + K103N + V108I; 10: K103N + V108I; 11a: K103N + V179I; 19, 20: K103N) only non-ETR RAMs were found.

Four samples from three patients had resistant HIV strains which showed highly increased IC_50_ values and a >15 fold decrease in susceptibility (5a: 45.8 µM; 5b: 71.4 µM; 13: 68.8 µM 14: 29.1 µM), while direct sequencing showed mutations which are not known to be predictive for decreased sensitivity for ETR (5a and 5b: K101I/R + V106G; 13: K103N + G190A; 14: A98S + K103N + E138A).

A further five samples (2, 3b, 6, 8a, 8b) had a >15 fold decrease in susceptibility and strongly increased IC_50_ (>100 uM). The mutational patterns were, to varying degrees, predictive of a decreased sensitivity to ETR, including Y181C (3b, 8a, 8b), V901 + L100I + K103N (6), and A98S + E138E/Q + K238T (2).

### Ultra-deep pyrosequencing (UDPS)

UDPS was performed on nine samples (1, 2, 5b, 10, 11a, 13, 14, 19, 20) for which there seemed to be a discrepancy between the major genotype and the RT phenotype (Table 3). The median nucleotide coverage per sample ranged from 1499 to 2506 sequences. Relative to UDPS, direct sequencing detected all mutations corresponding to >20% but no mutations <20% of the viral population. Altogether eleven mutations were detected by UDPS, but not by direct sequencing, ranging from 0.54% to 19.56%.

**Table 3:** NNRTI-resistance results obtained through phenotypic testing, direct sequencing and ultra-deep sequencing (UDPS).

	aFold			Mutations at amino acid*											Antiviral score according to		
Patient/Sample	difference in ETR IC_50_		90 V	98 A	100 L	101 K	103 K	106 V	108 V	138 E	179 V	181 Y	190 G	238 K	Stan	Tibo	Mono
1	11.8	Direct		S				A							0 (s)	0 (s)	2 (s)
		UDPS		S			T	A	I		I	C			30 (i)	2.5 (i)	7 (r)
		**2409(1127–3244)		*68.63%			18.8%	66.58%	0.54%		0.57%	6.88%					
2	>100	Direct					N			Q				T	10 (s)	1 (s)	2 (s)
		UDPS					N			Q				T	10 (s)	1 (s)	2 (s)
		1958 (1108–2870)					99.2%			32.47%				98.64%			
5b	71.4	Direct				I/R		G							0 (s)	0 (s)	0 (s)
		UDPS				I/R		G							0 (s)	0 (s)	0 (s)
		2305(1201–3397)				65.64%/31.09%		99.83%									
10	13.6	Direct					N		I						0 (s)	0 (s)	0 (s)
		UDPS					N		I		I				0 (s)	0 (s)	1 (s)
		1704(876–2325)					98.85%		100%		3.24%						
11a	7.3	Direct					N				I				0 (s)	0 (s)	1 (s)
		UDPS					N				I		A		10 (s)	1 (s)	2 (s)
		1613(873–1887)					97.44%				100%		3.59%				
13	68.8	Direct					N						A		10 (s)	1 (s)	1 (s)
		UDPS	I		I		N						A		25 (i)	6 (r)	4.5 (r)
		2506(1546–2696)	2.26%		0.61%		99.41%						95.57%				
14	29.1	Direct		S			N			A					5 (s)	1.5 (s)	3 (s)
		UDPS		S		E	N			A				T	15 (i)	2.5 (i)	6 (r)
		1855(1118–2536)		96.77%		0.81%	22.1%			100%				19.56%			
19	6.4	Direct					N								0 (s)	0 (s)	0 (s)
		UDPS					N/S	I							0 (s)	1.5 (s)	2 (s)
		1499(832–2325)					95.07%/3.05%	1.09%									
20	7.2	Direct					N								0 (s)	0 (s)	0 (s)
		UDPS					N								0 (s)	0 (s)	0 (s)
		2412(1228–2953)					97.37%										

ETR  =  etravirine; Direct  =  Direct Sanger sequencing; UDPS  =  Ultra-Deep Pyrosequencing; Stan  =  Stanford; Tibo  =  Tibotec; Mono  =  Monogram. *figure beneath amino acid indicate percentage consisting of a mutated population as determined by UDPS.

** Median (IQR) nucleotide coverage per patient.

a Fold difference compared to IC_50_ value of BH10-wild-type (1.0±0.18 µM), which was included in every run. A fold-change of <5 was considered as sensitive, between 5–15 as decreased susceptibility, and above 15 as resistant. A Stanford score of 0-<15, ≥15-<60 and ≥60 are defined as susceptible (s), intermediate (i) and resistant (r). Tibotec weighted genotypic score of 0–2, 2.5–3.5 and ≥4 are predictive of susceptible (s), intermediate (i) and reduced response (r). Monogram defines a weighted score of 0–3 as susceptible (s) and ≥4 as resistant (r).

In four samples (1, 11a, 13, 14), UDPS detected additional minor variants associated with decreased ETR susceptibility (1: Y181C; 11a: G190A; 13: L100I; 14: K101E + T238K).

In three samples (2, 5b, 20), identical mutations were found with the two sequencing techniques. Thus, the UDPS did not reveal any further minor variants that could explain the increased IC_50_ values of 100 µM, 71.4 µM and 7.2 µM, respectively.

In two samples (10 and 19), UDPS showed additional minor mutations not known to be associated with decreased ETR susceptibility (10: V179I; 19; V106I).

### Correlation between RT-based phenotypic sensitivity and genotype scoring based on direct sequencing

There was a significant correlation between the Stanford scoring system and the phenotypic assay results (r = 0.70 p<0.0001) assessed by Spearman's rank test. The Monogram and Tibotec scoring systems also correlated with the IC_50_ values (r = 0.65 p<0.0005; r = 0.64 p<0.0005, respectively).

### Resistance results in patients who had stopped NNRTI

Twelve samples (1, 3a, 6, 7, 9a, 9b, 11b, 12, 14, 15, 17, 20) of eleven patients were drawn after that an NNRTI containing regimen had been terminated (Tables 1 and 2). In patients 1 and 20, NVP and EFV had been stopped six and eight weeks earlier, respectively. The IC_50_ values were slightly increased (11.8 µM and 7.2 µM, respectively) but the Stanford scoring predicted full sensitivity (A98S + V106A; K103N, respectively). However, in patient 1 UDPS revealed additional minor mutations (K103T+ V108I + V179I + Y181C) which possibly could explain this difference (Table 3).

In patients 6 and 14, a clear decreased phenotypic sensitivity against ETR (6: >100 µM; 14: 28.8 µM) was found despite that >1 year and >3 years, respectively, had passed since the cessation of an NNRTI. Genotyping further supported this finding for patient 6 (direct sequencing: V90I + L100I + K103N) and patient 14 (UDPS: A98S + K101E + K103N + E138A + K238T).

In four patients, several years had passed since their NNRTI treatment was terminated (7, 11b, 15: >5.5 years; 9a: 3.5 years). In patient 12, 18 weeks had passed. No phenotypic resistance was found in these patients although non-ETR RAMs persisted (11b: V179I; 12 and 15: K103N; 9a: A98S + K103N + V108I + K238T). However, a second sample (9b) of patient 9 drawn four months later showed a slightly decreased phenotypic sensitivity (9.2 µM) with the same mutational pattern with a concomitant increase in plasma viral load. UDPS could not be performed due to lack of plasma.

### Resistance results in patients with ongoing NNRTI failure

Eight samples (3b, 5a, 5b, 8a, 8b, 10, 11a, 18) of six patients were drawn during failure of an ongoing NNRTI containing regimen. In four samples (3b, 8a, 8b, 18), a concordance was seen between the pheno- and genotypes. Thus, patient 3b exhibited a high ETR resistance with both methods (IC_50_ >100 µM; genotype: V90I + A98S + V179D + Y181C). In both samples of patient 8, phenotypic resistance (>100 µM) and the mutations K103N + Y181C were found. Patient 18 had a sensitive phenotype (4.1 µM) and was devoid of mutations other than K103N + V90I after 10 weeks failure.

In four samples, discordance was seen between the RT-based phenotype and the genotype. In patient 5, two samples were drawn with three months interval during failure with EFV-containing regimen. An increasing IC_50_ of 45.8 µM and 71.4 µM, respectively, was seen despite that direct sequencing as well as UDPS showed only mutations (K101I/R + V106G) which are not known to be ETR-associated. Patient 10 exhibited an increased IC_50_ (13.6 µM), but the identified mutations (direct sequencing: K103N + V108I; UDPS: K103N + V108I + V179I) predicted an ETR sensitive virus. In patient 11 a sample drawn early after failure showed a slightly increased IC_50_ (7.3 µM), while direct sequencing showed K103N + V179I. However UDPS identified additionally G190A giving support for that resistance was developing.

### Effects of ETR on patients with transmitted NNRTI resistance

Four patients (no 2, 13, 16, 19), who had been infected with an NNRTI resistant strain, were also analysed. Patients 2 and 13 had a strongly increased IC_50_ values (>100 µM; 68.8 µM) despite that the sequence analysis predicted only a slightly decreased sensitivity (2: K103N + E138Q, K238T; 13: K103N + G190A) (Table 2). The remaining two samples had the K103N only, and a low 1.7 µM (16) and a slightly increased 6.4 µM (19) IC_50_ value, respectively, was found. Patient 4 who had a K103R mutation was also sensitive with the phenotypic assay.

## Discussion

In high-income countries, there has been a considerable decline of HIV-1 drug resistance during the last decade [25], [26]. In contrast, transmitted and acquired HIV drug resistance is increasing in low-income countries [27], [28], especially resistance to the first generation NNRTIs, NVP and EFV [29]-[31]. The second generation NNRTI, etravirine (ETR), is approved for treatment-experienced patients and cross-resistance to NVP and EFV is considered to be limited. We have earlier shown that a simple and affordable RT-based phenotypic resistance test can be used for detection of ETR resistance, including cross-resistance to other NNRTIs in clinical samples. In this study, we further expanded the evaluation of the method in a larger clinical cohort including patients with past or ongoing failure on the first generation NNRTIs by comparing the results with those of direct sequencing and UDPS.

We found that most of strains from patients with ongoing or past failure to a first generation NNRTI had various degrees of decreased ETR susceptibility by our phenotypic test, despite that the mutational pattern based on direct sequencing did not always predict ETR resistance. For our assay, we previously demonstrated low IC_50_ values (mean ±SD: 2.5±1.0 µM) in treatment-naïve patients, a good reproducibility and an independence on the amount of plasma HIV RNA for the outcome of the test [22]. It can be argued that to describe a more precise laboratory cut-off for decreased susceptibility an extensive evaluation has to be done and that the assay may overestimate the presence of decreased susceptibility to ETR. However, in the present experiments ETR gained expected changes in IC_50_ values for all recombinant HIV-1 RTs equivalent to previous reports [5], [20], one NNRTI naïve patient (no 4) had a low IC_50_ value, and the test could plausibly describe the lack of impact of K103N on the IC_50_ value of ETR in the majority of samples [2], [5]. Thus, it is clear that the method can discriminate between mutants with or without ETR-relevant mutations.

One possible explanation for the discrepancy between the phenotypic and the genotypic test results obtained by direct sequencing is resistance in minor viral variants. Therefore, UDPS was used in nine samples in which a discrepancy was found. There was a concordance between the direct sequencing and the UDPS for mutations consisting of >20% of the viral population. Also, eleven additional RAMs were found by UDPS, in all cases <20% of the viral population, which is well in line with earlier results on the detection levels of direct sequencing [32]–[35]. Importantly, in four patients ETR RAMs were found in the minor quasispecies. In at least three of them, the proportion of mutated virus was so high that it is possible that these mutations may have contributed to the phenotypic resistance explaining the discrepancy with the genotype obtained by direct sequencing. Thus, it is possible that our phenotypic method is more sensitive than direct sequencing in identifying minor quasispecies with RAMs in the RT. The degree and pattern of an increased sensitivity as well as the clinical relevance remains to be determined.

Also, the clinical treatment history was concordant with our phenotypic results, which corresponded to a true decreased susceptibility for ETR. Thus, during early ART failure and before the NNRTI was stopped, an increase of the IC_50_ was seen in four of eight samples despite that a sensitive virus was predicted by the genotype. Also, in two patients who stopped NNRTI some weeks before the sampling, an increased IC_50_ but not ETR-resistance mutations was found. For subjects who had stopped the 1^st^ generation NNRTI-containing regimen for one or more years, a good concordance between the methods was seen. In addition, in two subjects (7 and 16) with very poor adherence, no phenotypic resistance was identified and only K103N in one of them. These patient histories and the UDPS comparison indicate that our phenotypic method may detect resistance to ETR despite that the direct sequencing predicts a sensitive phenotype.

The cause of the discrepancies between the phenotype and the genotype results is not known presently. The mutations predictive for cross-resistance to ETR-resistance have been mainly identified in clinical studies using direct sequencing [36] and it cannot be excluded that not-yet described mutations exist which may influence the ETR sensitivity. The effects of the defined mutations may also vary in different genetic environments. It seems less likely that the replicative capacity of the virus influenced the results although the ratio of relative RT activity to infectious titer has been reported to group according to virus tropism [37]. Thus, there was no correlation between the viral load in plasma, the CD4 T-cell counts and the IC_50_ of the phenotypic test (data not shown). Also, we have previously shown that the RT amount can vary within a range of 40-828 fg RT/ml without affecting the determination and reproducibility of IC_50_ values [22]. It should be remembered that our phenotypic method uses lysates originating from intact virions. In contrast, the genotypic and phenotypic methods based on recombinant viruses use HIV RNA which partly may represent defective virus [37], [38]. Actually, it has been reported that endogenous RT activity, not p24 content or viral RNA load, is the best surrogate measure of infectious HIV-1 titer in both cell-free supernatants and viruses purified on sucrose cushions [37]. It can therefore be speculated that the viral population which is studied with our phenotypic assay is more representative for the ongoing viral replication.

The clinical utility of the phenotypic method remains to be established. It is clear that there is a strong correlation between the results of our phenotypic method and the predicted antiviral scores according to three genotypic scoring systems. A high IC_50_ was found in all samples with key-mutations, Y181C and L100I, which are associated to ETR resistance. However, in a substantial number of samples there was a phenotypically decreased susceptibility but no known ETR-RAMs. The clinical relevance of these findings and clinical cut off of our method should be studied on larger patient-populations. However, in view of the high potency of newer anti-HIV drugs and the low failure rate of todays ART, such a study is likely to demand samples from a larger international study.

In the present perspective, it is believed that the RT-based phenotypic method for detection of ETR resistance in plasma is possible it could be used as an alternative for studying resistance to first and second generation NNRTI. While waiting for studies on its clinical usefulness, it could also be useful for studies on the kinetics of NNRTI resistance during ART failure.
